# Editorial: Emergentist Approaches to Language

**DOI:** 10.3389/fpsyg.2021.833160

**Published:** 2022-01-20

**Authors:** Brian MacWhinney, Vera Kempe, Patricia J. Brooks, Ping Li

**Affiliations:** ^1^Department of Psychology, Carnegie Mellon University, Pittsburgh, PA, United States; ^2^Division of Psychology, Abertay University, Dundee, United Kingdom; ^3^Department of Psychology, College of Staten Island and the Graduate Center, CUNY, Staten Island, NY, United States; ^4^Faculty of Humanities, Hong Kong Polytechnic University, Kowloon, Hong Kong SAR, China

**Keywords:** emergentism, corpora, phonology, crosslinguistic, constructions, child language, second language acquisition

The articles in this Frontiers special issue use the analytic and theoretical tools of emergentism to explain a wide variety of language structures and processes. Early discussions of emergentism were provided by Lewes (1877), Bergson (1907), and Alexander (1920) with extensions into the frameworks of General Systems Theory by von Bertalanffy (1968), autopoiesis by Varela et al. (1974), and dynamic systems theory by Thelen and Smith (1994). Emergentist thinking has been entrenched in the biological and physical sciences for well over a century. Darwin (1859) explained the shape of the beaks of the finches of the Galapagos as emerging from adaptation to the constraints of available food sources which are in turn shaped by varying weather patterns across the islands. Geologists explain the structure of mountain ranges and ocean rifts as emerging from the constraints of crustal plate movements which are in turn shaped by processes in the mantle. However, language scientists have made little use of emergentism, often focusing on description of language structures, rather than on explanations of how those structures arise. Emergentism provides a way of moving language studies forward by going beyond description to explanation.

In its application to human language, emergentism focuses on three core analytic frameworks: competition, structural levels, and time/process frames (MacWhinney, 2015). Regarding competition, the theory builds on Darwin's linking of evolution and adaptation to the operation of proliferation, competition, and selection. In language, we see proliferation in dialect and speaker variation, semantic drift, construction generalization, and languages in contact. The competition between these many form-function mappings then impacts all structural levels from articulation up to conceptualization and code-switching. Regarding structural levels, linguistic theory has identified patterns on the levels of audition, articulation, lexicon, morphology, syntax, discourse, narrative, and conversation. On each of these levels, we find that structures emerge from the impact of constraints on possible forms. Regarding time/process frames, emergentist theory shows how structures emerge from constraints applying uniquely to the online time/process frames of neural transmission, auditory processing, articulation, lexical linearization, self-monitoring, sentence planning, mental model construction, intention formation, conversational turn-taking, and code-switching. In addition, structures emerge from constraints on the longer time/process frames of memory consolidation, rehearsal, statistical learning, development, parental support, social group attachment, professional specialization, dialect shift, historical change, language evolution, second language learning, and bilingual representation (see, for example, Hernandez et al., 2005). The greatest current challenge to emergentist theory is to understand quantitatively how forces and constraints on these many different time/process frames work upon competing alternatives to shape language structure. The papers in this special issue each advance specific aspects of this larger emergentist theory.

1. The two papers in the first section conceptualize language as a network, with direct mappings between forms and functions.

O'Grady applies three key ideas from emergentist theory to provide an account of linguistic coreference: (1) meanings map directly onto forms, without an intervening syntactic (tree) structure; (2) algorithms bring together forms and meanings during speech production and comprehension; (3) properties of algorithms are shaped by real time processing. He goes on to examine constraints on linguistic coreference in English and Balinese as providing evidence against a language-specific account of the syntax of anaphora and in support of the emergentist approach. This work sheds light on how emergentist approaches can account for universal patterns and similarities across typologically different languages.

Diessel proposes that linguistic structure emerges from a dynamic network of associations comprising symbolic (i.e., associations between forms and meanings), sequential (i.e., associations between elements in sequences), and taxonomic relations (i.e., associations between representations at different levels of abstraction). Other papers in this volume similarly adopt the framework of network science in studies of monolingual (Chan et al.) and bilingual (Xu et al.) language acquisition.

2. The four papers in the second section provide illustrations of how emergentist ideas may be implemented across different time/process frames, ranging from historical change to online processing.

Goldberg and Lee explore diachronic changes in the preferred order of gendered binomial expressions in English using large-scale data from the Google N-gram online corpus. They document how drastically asymmetric word frequencies made the binomial expression *mother and dad(dy)* more cognitively accessible than *dad(dy*) *and mother*. As the emergent cluster of semantically and morphologically related binomials (e.g., *ma and pa*; *grandma and grandpa*) grew in strength over time, it progressively coaxed other binomials to shift order, with larger effects in promoting female-first binomials against less entrenched male-first competitors. Their analysis provides a novel demonstration of how the ease with which information is retrieved from long-term memory not only influences language processing (MacDonald, 2013), but also results in historical change.

Sagae illustrates how a computational learning model that makes use of new algorithms for “deep learning” can provide an accurate estimate of the developmental level for a child's syntax. This model can learn from transcripts alone with no prespecified language knowledge and without reliance on corrective feedback. Remarkably, this naive emergentist model does just as well as hand-crafted and hand-computed models, thereby illustrating how researchers can pursue a fully data-driven approach to understanding language development and in conducting clinical assessments.

Gow et al. use neuroimaging to explore how learning words with illegal phonotactic sequences influences phonological processing. Updating of phonotactic constraints as a consequence of word learning was associated with co-activation of brain regions that support lexical word-form representations as opposed to areas involved in rule-based processing. The findings align with predictions of the TRACE connectionist model of speech perception (McClelland and Elman, 1986), emphasizing top-down influences of lexical representations on phonological processing and emergence of phonotactic constraints out of the structure of the lexicon.

Yang et al. examine three different accounts for why there are syntactic priming effects: (1) the transient activation account focuses on the role of reactivation of declarative memory structures (Branigan et al., 2000); (2) the error-based implicit learning account places emphasis on the speaker's prediction errors while processing sentences (Bock and Griffin, 2000); and (3) the reinforcement learning account highlights feedback signals on procedural knowledge (Sutton and Barto, 1998). The three accounts make different assumptions regarding the representation of syntactic rules (declarative vs. procedural) and the mechanisms that drive priming. Through a series of computational models implemented in ACT-R (Anderson et al., 2004), the authors tease apart the role of different mechanisms in each account and conclude that the data are largely consistent with the error-based implicit learning account.

3. The five papers in the third section use emergentist concepts to explain patterns in children's learning of phonological and grammatical structures.

Menn et al. introduce a new CHILDES corpus of phonetically transcribed dialogue involving a child and his communicative partners, spanning the child's babbling and first words to well-developed single word communications. The authors present analyses illustrating how the child's first words surface from poorly coordinated articulatory gestures, and how various articulatory units (phonetic segments, syllables, words) emerge gradually over time. The detailed transcriptions of the corpus enable the authors to trace variability in the child's speech productions to variability in the realization of the various articulatory components which shape the non-linear trajectory of segmental development.

Rose and Penney examine how constraints from different structural levels impact children's learning of consonantal place and manner features. These levels include specifics of the articulatory process, variations in dialect, the shape of the child's lexicon, and the overall segmental inventory of the language. They illustrate the interactions of these constraints in terms of data on the late acquisition of rhotic consonants across children and languages and on a case study of a German child's substitutions of labials for coronals. The overall perspective of this work is that “segments are made, not born” in that both the sequence of acquisition and the shape of the emerging system is determined by the interaction of constraints from several levels.

Tsung and Gong use data from 168 children in the Early Childhood Mandarin Corpus to track the emergence of distinct varieties (11 types) of the Mandarin “ba” construction in child language, and children's knowledge of various constraints associated with its use. An important finding in this study is that children's linguistic development is constrained by and co-evolves with pragmatic acquisition and other non-linguistic cognitive factors, and that these developments tend to come in dynamic waves and stages. This paper, along with others in this volume (e.g., Goldberg and Lee; Menn et al.; Zhao and Fan), demonstrates the utility and significance of corpus analysis in the study of emergentism in language processes.

Donnelly and Kidd test predictions of two models of syntactic development—early abstraction and usage-basis—through use of the intermodal preferential looking paradigm (Golinkoff et al., 2013). The authors find that 21-month-old English-speaking toddlers do not have a sufficiently robust representation of the transitive construction to support comprehension of novel verbs. Using statistical models that assume either graded or discrete individual differences, the authors fail to find conclusive support for either theoretical approach and conclude that existing experimental paradigms might be ill-suited for studies of individual differences.

Chan et al. use an elicited-production task to explore Cantonese-speaking children's earliest relative clauses. Building on research on “construction conspiracy” (Abbot-Smith and Behrens, 2006) and “constructional grounding” (Israel et al., 2000), the authors explore whether the similarity of a new construction to existing structures in the child's repertoire (or network) promotes acquisition to a greater extent than structural complexity. In the case of Cantonese, object-relative clauses resemble the high frequency Subject-Verb-Object construction but are arguably more complex than subject-relative clauses. Though the children produced few relative clauses, their elicited productions favored object-relative clauses, supporting the emergentist view that learning is guided by similarities to known, high frequency constructions.

4. The three papers in the fourth section explore domain-general mechanisms underlying language abilities and shared processing resources that yield cross-domain effects. These mechanisms support sensorimotor coordination, learning of serial order and co-occurrence statistics, and priming.

Tkachman et al. suggest that the central pattern generators that drive locomotion also constrain the structure of bimanual actions in sign languages. They present evidence from multiple unrelated sign languages, indicating that bimanual signs with alternating movements are more likely to involve repetition than bimanual signs that are symmetrical. They argue that the motor behaviors evolved for non-linguistic purposes may be exploited for linguistic purposes, specifically in relation to the emergence of sign language conventions.

Koranda et al. use interleaved syntactic priming and action priming tasks to explore the hypothesis that planning in the service of language production emerges from domain-general action planning processes. Findings of this type indicate how patterns can emerge from constraints from very disparate systems when they are interacting online.

Lahti-Nuuttila et al. consider whether children with Developmental Language Disorder (DLD) exhibit a domain-general impairment in short-term memory (STM) for serial order. Exploring links between non-linguistic auditory and visual STM and receptive and expressive language in typically developing children and children with DLD, they find that non-verbal STM for serial order modulates aspects of language development in children with DLD. The results point to how serial processing can impact language when the state of acquisition is less developed and more fragile.

5. The five papers in the fifth and final section use large-scale corpora to study second language learning of English and bilingual language processing. This includes testing the impact of the Competition Model dimensions of cue availability/reliability (MacWhinney, 2021) and dominant contextual patterns on processing.

Zhao and Fan use written corpus data to test predictions of the Competition Model in the context of Chinese university students' acquisition of English article constructions. In structural equation models predicting accurate production of English articles, learners at all levels of proficiency were influenced by cue availability whereas only higher proficiency learners were influenced by cue reliability. A future direction in this work is to link the Competition Model to a usage-based account of acquisition and production of constructions such as the English particle.

Guo and Ellis use an elicited imitation task to explore influences of cue availability, cue reliability, and phrasal formulaicity on L2 learners' production of English inflectional morphemes. Learners were more likely to produce morphemes in words that reliably occur in the target inflected form and in high frequency multi-word strings. This suggests that the emergent features of the input facilitating learning and accurate production of grammatical morphemes exist at multiple levels of granularity.

Zeng et al. explore how prototypical associations between lexical-semantic features and grammatical forms influence English tense-aspect processing in L2 learners varying in proficiency. They document how lexical-semantic features of regularly inflected verbs affect online sentence processing and offer a statistical learning account of how frequencies of correlated features shape both online processing and L2 acquisition.

Evans and Larsen-Freeman apply dynamic systems theory to L2 acquisition of *before-* and *without-*headed adverbial constructions. Using a longitudinal case-study design, the authors observe how non-linear developmental trajectories emerge from the dynamics of competition between specific forms and functions.

Xu et al. examine corpora of naturalistic (spontaneous) code-switching in English-Chinese and English-Spanish bilinguals through the lens of network science. Traditional psycholinguistic studies of code-switching rely on experimenter-induced code-switching behaviors, and account for why code-switching occurs through lexical accessibility and psycholinguistic factors. The authors examine the global lexico-semantic structures of words that occur in naturalistic code-switching and identify how network communities and clustering coefficients could depict the impact of bilingual lexical properties on bilingual production in a holistic manner. The study offers a new way to look at the organization and competition of bilingual lexical representation and processing.

## Conclusions

Human language structure is remarkably complex and dynamic. This complexity arises from a myriad of social, cognitive, and biological forces competing across diverse timescales and within many processes. The analyses and findings presented here have shown how one can trace these interactions, competitions, and constraints to observe the emergence of linguistic structures in phonology, lexicon, and grammar. These emergentist studies have used corpus analysis, experimentation, neuroimaging, and computational modeling to study the determination of language structure across the timescales of processing, development, and language change.

Some studies, such as Koranda et al., emphasize the ways in which linked systems constrain each other in real time. Others, such as Rose and Penney, show how constraints across levels shape children's learning, whereas studies such as Evans and Larsen-Freeman show how this shapes adults' learning across months and years. Still others, such as Goldberg and Lee, show how repeated patterns of preferential processing bring about historical shifts in language structure.

The work reported here helps us understand the ways in which competition across levels and timeframes works within processes to shape structures. We have rigorous methodologies to test these accounts, and not all emergentist accounts will stand up to these tests. However, the failures can then lead us to consider a broader set of constraints and mechanisms that will allow our models to better match the results of our corpus analyses and experiments. Interestingly, work in Generative Grammar has also begun to focus on the emergence of structure from constraints operative across varying timescales (Fitch, 2014; Newmeyer, 2017; Watumull and Chomsky, 2020) and with language-external constraints (Chomsky, 2005, pp. 9–10), pointing to potential bridges between competing theoretical perspectives. In these ways and many others, emergentist thinking is opening exciting new pathways for understanding language.

## Author Contributions

All authors listed have made a substantial, direct, and intellectual contribution to the work and approved it for publication.

## Funding

This work was supported by symposium grant R13DC018182 from NIDCD and NICHD titled TalkBank for the Next Generation.

## Conflict of Interest

The authors declare that the research was conducted in the absence of any commercial or financial relationships that could be construed as a potential conflict of interest.

## Publisher's Note

All claims expressed in this article are solely those of the authors and do not necessarily represent those of their affiliated organizations, or those of the publisher, the editors and the reviewers. Any product that may be evaluated in this article, or claim that may be made by its manufacturer, is not guaranteed or endorsed by the publisher.
